# The Role of Specialized Pro-Resolving Lipid Mediators in Inflammation-Induced Carcinogenesis

**DOI:** 10.3390/ijms241612623

**Published:** 2023-08-10

**Authors:** Wheeler Torres, José Luis Pérez, María P. Díaz, Luis D’Marco, Ana Checa-Ros, Rubén Carrasquero, Lissé Angarita, Yosselin Gómez, Maricarmen Chacín, Paola Ramírez, Nelson Villasmil, Samuel Durán-Agüero, Clímaco Cano, Valmore Bermúdez

**Affiliations:** 1 Endocrine and Metabolic Diseases Research Center, School of Medicine, University of Zulia, Maracaibo 4004, Venezuela; 2 Grupo de Investigación en Enfermedades Cardiorrenales y Metabólicas, Departamento de Medicina y Cirugía, Facultad de Ciencias de la Salud, Universidad Cardenal Herrera-CEU, CEU Universities, 46115 Valencia, Spain; 3 Escuela de Nutrición y Dietética, Facultad de Medicina, Universidad Andres Bello, Concepción 4260000, Chile; 4 Facultad de Ciencias de la Salud, Universidad Simón Bolívar, Barranquilla 080022, Colombia; 5 Facultad de Ciencias Para el Cuidado de la Salud, Universidad San Sebastián, Los Leones 8420524, Chile

**Keywords:** cancer, carcinogenesis, chronic inflammation, specialized pro-resolving lipid mediators, bioactive lipids, polyunsaturated fatty acids

## Abstract

Cancer is a process involving cell mutation, increased proliferation, invasion, and metastasis. Over the years, this condition has represented one of the most concerning health problems worldwide due to its significant morbidity and mortality. At present, the incidence of cancer continues to grow exponentially. Thus, it is imperative to open new avenues in cancer research to understand the molecular changes driving DNA transformation, cell-to-cell interaction derangements, and immune system surveillance decay. In this regard, evidence supports the relationship between chronic inflammation and cancer. In light of this, a group of bioactive lipids derived from polyunsaturated fatty acids (PUFAs) may have a position as novel anti-inflammatory molecules known as the specialized pro-resolving mediators (SPMs), a group of pro-resolutive inflammation agents that could improve the anti-tumor immunity. These molecules have the potential role of chemopreventive and therapeutic agents for various cancer types, and their effects have been documented in the scientific literature. Thus, this review objective centers around understanding the effect of SPMs on carcinogenesis and their potential therapeutic effect.

## 1. Introduction

Cancer is one of the most devastating public health problems worldwide due to its significant morbidity and mortality [1]. Although the risk of developing cancer has increased, the mortality rates in a significant fraction of cancer types have decreased considerably in the last two decades, partly by introducing new and more effective screening techniques, leading to a higher tumor detection rates at early stages [2,3]. The other battlefield to control this epidemic is to develop more effective, specific, and less toxic antineoplastic agents. This movement has been a successful strategy that has paid off over the past three decades, which have seen the emergence of several new agents termed targeted antineoplastics. Monoclonal antibodies [4], tyrosine kinase inhibitors [5], mTOR inhibitors [6], retinoids [7], immunomodulatory agents (IMiDs) [8], enzymes and CRISP-R technology [9], BRAF kinase inhibitors [10], BCG vaccine [11], and phosphodiesterase-3 inhibitors [12], among others, are examples of drugs that have changed the paradigm of traditional cancer management.

Cancer is the second leading cause of death globally, and despite the aforementioned technological innovations, the cancer burden had risen to 18.1 million new cases and 9.6 million deaths in 2018 [13]. Thus, according to the World Health Organization’s International Agency for Research on Cancer Global Cancer Observatory (GLOBOCAN) forecast, 27.5 million new cancer cases worldwide are expected in 2040 [3,14]. In this context, demographic factors, such as population growth, aging, social behaviors, and economic development, will drive these projections [15]. However, the Prospective Urban Rural Epidemiology (PURE) study conducted on individuals 35–70 years of age from 21 countries worldwide has shifted our traditional view of cancer epidemiology, showing that deaths from cancer are higher when compared with cardiovascular disease (CVD) in high-income countries [16,17]. This study clearly shows a lower incidence of CVD deaths than cancer deaths when a country’s gross income stratum is high. Overall, a combination of screening, prevention, and successful treatment has resulted in a considerable increase in cancer survivors in the last five decades.

It is well known that cancer occurs due to multifactorial causes, with a substantial genetic component; however, nearly 25% of the origins of a variety of tumors are caused by chronic tissue inflammation [18,19]. In this vein, recent research has revealed that certain social, environmental, and lifestyle factors can promote chronic systemic inflammation that, in turn, can lead to several diseases, such as CVD, diabetes mellitus, chronic kidney disease, non-alcoholic fatty liver disease, autoimmune and neurodegenerative disorders, and cancer [20,21]. In this context, inflammation was first connected with cancer in 1828 by Jean Marjolin and later in 1833 by Caesar Hawkins, who described skin cancer lesions near burn wounds years after the heat damage. In 1863, Virchow was the first to hypothesize that cancer genesis relies on chronic inflammation via both irritation and injury-enhancing cell proliferation [22,23].

Today, the causal relationship of inflammation and innate immunity to cancer is more widely accepted; however, some of the molecular and cellular mechanisms mediating this relationship remain unresolved, but recent data have expanded our knowledge about the participation of inflammation as a critical component of tumor behavior. In this regard, inflammatory cells within the tumor niche are necessary for proliferation, survival, and migration enhancement. In addition, tumor cells can express some signaling molecules from the innate immune system, such as chemokines, selectins, and their receptors for invasion, migration, and metastasis, amplifying the process mentioned above. These processes open new avenues by new anti-inflammatory approaches with direct anti-cancer effects or even with the ability to interfere in normal-to-neoplastic cell transformation within a highly inflammated niche. Therefore, it is still fundamental to study the close link among these entities, where the transformation of damaged DNA, the changes in lipid and protein metabolism, and the production of free radicals constitute critical processes of carcinogenesis [24,25,26].

Therefore, cancer research has continued to look relentlessly for alternatives that can improve these therapies and offer fewer toxic events [27,28]. This fact is precisely one of the focal points of conventional cancer treatment, which involves considerable adverse effects despite having favorable results. Hence, with the emergence of molecular lipidomics, four novel lipid families have been recently discovered: Resolvins (Rvs), Maresins (MaRs), Protectins (PDs), and Lipoxins (LXs), belonging to a large group of molecules known as *The Specialized Pro-resolving Lipid Mediators* (SPMs) [29,30,31]. These compounds have been well-characterized since their identification as potent modulators of the immune response and for their effects on inflammation resolution. Furthermore, they have a potential effect on anti-tumor immunity [32,33]. For this reason, the objective of this review centers around the understanding of SPMs effects on carcinogenesis and their potential therapeutic effect. 

## 2. Specialized Pro-Resolving Lipid Mediators: An Overview

### 2.1. Origin, Biosynthesis, and Classification

**Figure 1:**
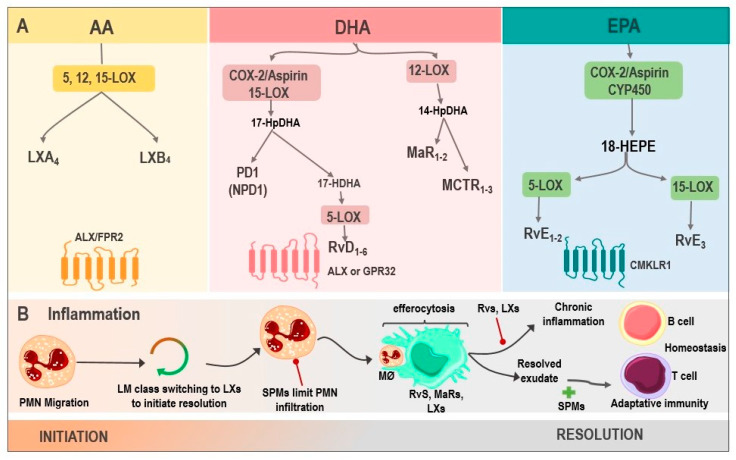
Biosynthesis of SPMs and their actions in inflammation. (**A**) LX are generated from AA via 5, 12, 15-LOX, resulting in LXA4 and LXB4, whose receptor is ALX/FPR2. DHA, via COX-2/Aspirin as well as via 15-LOX, produces 17-HpDHA, which can be metabolized into PD1 or NPD1 and is synthesized in the nervous system or into 17-HDHA, generating RvD1-6 via 5-LOX, with activity on ALX or GPR32. Another DHA pathway is through 12-LOX, where MaR1-2 and MCTRs are produced. Finally, the biosynthesis of RvE1-3 derives from EPA, by the enzymes COX-2/Aspirin or CYP450, being their receptor CMKLR1. (**B**). SPMs in inflammation: the inflammatory microenvironment starts with PMN migration. Afterward, the change of pro-inflammatory LMs into pro-resolving ones occurs with the initial synthesis of LXs. PMN infiltration increases, and SPMs act at this point, reducing this influx. Moreover, the efferocytosis by MØ is stimulated and improved by Rvs, MaRs, and LXs. Adaptive immunity, stimulated by SPMs, participates during the final phase of resolution. However, whenever there is an exaggerated and chronic inflammatory response, it leads to chronic inflammation, inhibited by Rvs, and LXs. Abbreviations: SPMs: specialized pro-resolving mediator; AA: Arachidonic acid; LXs: Lipoxins; LOX: lipoxygenase; LXA4: lipoxin A4; LXB4: lipoxin B4; ALX: G protein-coupled lipoxin A4 receptor; formyl peptide receptor; DHA: docosahexaenoic acid; COX-2/Aspirin: Aspirin acetylates cyclooxygenase-2; 17-HpDHA: 17-hydroperoxyDHA; PD1: Protectin 1; NPD1: neuroprotectin 1; 17-HDHA: 17-hydroxy-DHA; Rvs: D1-6-series resolvins; GPR32: G protein-coupled receptor; MaRs1-2: Maresins 1-2; MCTR: maresin conjugates in tissue regeneration; RvE1-3: E1-3-series resolvins; EPA: Eicosapentaenoic acid; CYP450: cytochrome P450; CMKLR1: chemokine-like receptor 1; PMN: polymorphonuclear neutrophil; LM: lipid mediators; and MØ: macrophages.

For decades, immunopathology has faced the challenge of elucidating the mechanisms of associating chronic disorders with inflammation. Within the pleiad of molecular pathways, mediators, receptors, and metabolites, the resolution of inflammation has been a well-recognized process where complex signaling downregulates the immune cascade once the noxious stimulus is eliminated by both innate and adaptative immune responses and, as a result, the recovering of tissue homeostasis occurs [34]. Upon this premise, Serhan et al. [35] were the first to identify lipid metabolism products involved in this process by describing a family of bioactive compounds later coined SPMs (Figure 1), a group of molecules derived from omega-6 polyunsaturated fatty acids (ω-6 PUFAs) metabolism, such as arachidonic acid (AA; 20:4n − 6), and omega-3 polyunsaturated fatty acids (ω-3 PUFAs), such as eicosapentaenoic acid (EPA; 20:5n − 3) and docosahexaenoic acid (DHA; 22:6n − 3) via COX/LOX pathway [36].

SPMs belongs to G protein-coupled receptors ligands [37], and their critical role in regulating inflammation resolution has been widely documented, establishing that excessive or uncontrolled inflammation is tightly linked to a disbalance in their synthesis. Therefore, it is vital to understand every SPMs family’s role in the framework of acute and chronic inflammation [38].

### 2.2. Resolvins

Rvs are unique molecules synthesized from DHA and EPA by polymorphonuclear leukocytes (PMN) and macrophages during the resolution of inflammation to counter-regulate pro-inflammatory activity and promote efferocytosis [39]. In this regard, five resolvins families have been identified to date: the resolvin Ds (RvDs) 22-carbon DHA metabolites; the resolvin Es (RvEs) 20-carbon EPA metabolites; the resolvin Dn-6DPA (RvDsn-6DPA), a group of metabolites derived from the Osbond acid; the resolvin Dn-3DPA (RvDn-3DPA) that come from clupanodonic acid metabolism; and the resolvin Ts (RvTs), a clupanodonic acid metabolite [40].

RvDs are poly-hydroxyl metabolites of DHA, and to date, six RvDs (RvsD1-6), which vary in the number, position, and chirality of their hydroxyl residues and the position and cis-trans isomerism of their six double bonds, have been described [40]. Thus far, these variants have been identified in PMN and macrophages inflammatory exudates with a variety of allylic–epoxide intermediaries containing RvD1-2 at the start of the resolution, and later, RvD3-4 [41]. Furthermore, to carry out their functions, RvDs are ligands of G protein-coupled receptors (GPCR), such as ALX/FPR2, GPR18, GPR32, TRPA1, and the TRPV1 receptor [39].

On the other hand, EPA-derived E-series Rvs (RvE) includes four primary bioactive mediators (RvE1, RvE2, RvE3, and18-HEPE), synthesized by 5-LOX and 15-LOX in PMN and macrophages. These molecules exert their functions by Chem23, BLT1, and TRPV1 receptor-binding [42]. It has been reported that RvE1 regulates leukocyte adhesion molecules expression, ADP-dependent platelet activation, and PMN apoptosis stimulation [43]. In addition, RvE1, along with RvE2, increases IL-10 synthesis and phagocytosis [44]. Moreover, it is relevant to highlight the properties of RvE3 in the decrease in PMN and 18-HEPE production. These properties have been associated with a cardioprotective function in several studies since RvE1 protected against reperfusion injury in this open-chest rat model of ischemia–reperfusion [45] and protects against doxorubicin-induced cardiotoxicity by inhibiting oxidative stress, autophagy, and apoptosis by targeting AKT/mTOR signaling [46].

13-series Rvs (RvT) are also derived from ω-3 PUFAs, from docosapentaenoic acid (DPA), and synthesized by COX-2 during the resolution of acute inflammation. This family includes four mediators, RvT1, RvT2, RvT3, and RvT4 [24]. In this vein, Dalli et al. [47] described the anti-inflammatory role of RvsT1-4 in mice endothelial cells and neutrophil co-cultures during *E. coli* infection, demonstrating a protective response through the regulation of inflammation. Other important resolvins are the RvDsn-6DPA, a metabolite of the osbond acids, and resolvin Dn-3DPA (RvDn-3DPA), a clupanodonic acid metabolite. On the other hand, AT-RvDs are synthesized by non-native COX-2 (drug-modified cyclooxygenase 2) to form 17 (R)-hydroxyl residue named aspirin-triggered RvDs (AT-RvDs).

### 2.3. Lipoxins

LXs, synthesized in platelets and leukocytes from AA, are another group of bioactive lipids capable of carrying out a wide variety of functions. These include regulating the synthesis of pro-inflammatory cytokines, inhibiting angiogenesis, and reprogramming M2 macrophages by binding to ALX/FPR2 and GPR32 receptors [48]. Their variants, LXA4 derived from AA and aspirin-triggered LXA4 (ATL), are generated by 15-LOX. These compounds have protective anti-inflammatory actions on various physiologic and pathophysiologic processes as endogenous lipids acting in the resolution phase upon an inflammatory response [49], playing an essential role in the tumor microenvironment (TME) and cancer pathogenesis in several neoplasms, such as pancreatic, liver and colon cancer, melanoma, leukemia, and Kaposi’s sarcoma [50]. The healing role of these molecules has been widely demonstrated in preclinical studies. For instance, Madi et al. [51] evaluated LXA4 administration to mice with gastric ulcers, resulting in a significant histopathologic and immunohistochemical improvement in the gastric mucosa. 

### 2.4. Maresins

MaRs, also called macrophage mediators in resolving inflammation, are an SPMs family synthesized from DHA by the 12-LOX enzyme. These have functions related to phagocytes, such as the inhibition of neutrophil recruitment and macrophage efferocytosis stimulation [52]. Moreover, they can negatively regulate the synthesis of pro-inflammatory cytokines, such as IL-1β, IL-6, and TNF-α, to induce the resolution of inflammation and tissue regeneration [53]. The members of this family are maresin 1 (MaR1), maresin 2 (MaR2), and maresin conjugates in tissue regeneration (MCTR1-3). These compounds are synthesized by macrophages via lipooxygenation at the carbon-14 position with molecular oxygen insertion. Finally, MaRs action is achieved through BLT1 and TRPV1 receptors binding [54].

### 2.5. Protectins

The last group of SPMs are PDs, which, just like MaRs and RvD, are biosynthesized from DHA by the action of 15-LOX during the resolution of inflammation [55]. Protectin D1 (PD1) is the first and best-studied member of this family. This compound is synthesized by various cells, including leukocytes, such as PMN, eosinophils, and macrophages [56]. PD1 is also known as neuroprotectin D1 (NPD1) when synthesized in neural systems, and it has a protective action in the brain, retina, and in pain modulation [57]. Like other SPM family members, NPD1 exerts potent anti-inflammatory and anti-apoptotic/neuroprotective biological activities [58,59]. NPD1 actions in the central nervous system (CNS) are mediated by its interaction with the parkin-associated endothelin-like receptor (Pael-R), also known as GPR37, widely expressed in glial cells, such as astrocytes and oligodendrocytes [59].

Other neuroprotective structurally related agents exhibiting similar activity are PDX (10R, 17S-dihydroxy-4Z, 7Z, 11E, 13Z, 15E, 19Z-DHA); 20-hydroxy-PD1 (10R, 17S, 20-trihydroxy-4Z, 7Z, 11E, 13E, 15Z, 19Z-DHA); and 10-epi-PD1 (10R, 17S-Dihydroxy-4Z, 7Z, 11E, 13E, 15Z, 19Z-DHA) [60,61]. The beneficial effect of these anti-inflammatory mediators was reported by Sheets et al. [62], who demonstrated both a laser-induced choroidal neuro-vascularisation attenuation and microglia cells elongating in mouse eyes treated with NPD1. In addition, evidence in aged-mice models indicates that NPD1 is able to diminish post-operative delirium (POD). Post-surgical administration of NPD1 decreased IL-6 and TNF-α expression systemically as well as in the hippocampus and pre-frontal cortex; moreover, this SPM maintains the integrity of the blood–brain barrier (BBB) and induces macrophages polarization into an M2 phenotype, limiting neuroinflammation and cognitive decay [63].

## 3. From Inflammation to Cancer: A Key Topic in Carcinogenesis

Inflammation is a response to an acute process that possesses a therapeutic nature since it is a defensive mechanism against both external (e.g., bacteria and viruses) and internal agents (e.g., damaged cells and toxic compounds) [64,65]. Nevertheless, when it extends in time, it can become detrimental. Chronic inflammation is associated with various pathological entities, such as diabetes [66] and rheumatoid arthritis [67]. In fact, the existence of previous inflammatory diseases has shown an increased incidence risk for certain cancers. For instance, it has been reported that the incidence of colorectal cancer (CRC) is 4–10 times greater in individuals with ulcerative colitis (UC) in comparison with those without the inflammatory bowel disease [68]. Similarly, statistically significant evidence shows an association between prostate and ovarian cancer incidence, with the presence of previous inflammatory processes, such as prostatitis [69] and pelvic inflammatory disease [70], respectively.

**Figure 2:**
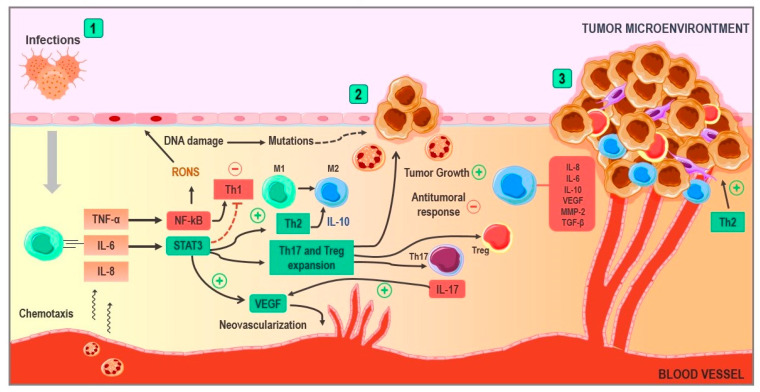
Implications of chronic inflammation in cancer development. (1) In situations such as persistent infections, an immune response is triggered, and this leads to the release of pro-inflammatory cytokines, such as TNF-α, IL-6, and IL-8, inducing the chemotaxis of immune cells and the production of free radicals. When the inflammatory process becomes chronic, the reactive oxygen and nitrogen species are capable of damaging DNA. TNF-α activates the NF-KB pathway, generating free radicals and a Th1 response with certain anti-tumor properties. However, the activation of the STAT-3 pathway by IL-6 counteracts this effect, generating a Th2 response and inducing the production of IL-10. (2) The STAT-3 pathway stimulates neovascularization via the production of VEGF, as well as through the stimulation of the expansion of Th17 and Treg lymphocytes. These lymphocytes contribute to tumor growth and neovascularization via IL-17 and inhibit the mechanisms of anti-tumoral immunity. The Th2 response generates IL-10, pushing macrophages towards the M2 phenotype known for their pro-tumoral properties, such as the release of substances that favor tumor growth and survival. (3) At this point, tumor growth is stimulated by pro-inflammatory cytokines in the environment. Angiogenesis continues at the expense of VEGF, and cytokines, such as IL-10 and TGF-B, contribute to the processes of immune evasion, generating a state of tumor-induced immunosuppression. Abbreviations: TNF-α: tumor necrosis factor Alpha; IL-6: Interleukin 6; IL-8: Interleukin 8; IL-10: Interleukin 10; IL-17: Interleukin 17; NF-κB: Nuclear factor kappa B; STAT3: Signal transducer and activator of transcription 3; Treg: Regulatory T Cell; VEGF: Vascular endothelial growth factor; TGF-β: Transforming growth factor beta; and MMP-2: matrix metalloproteinase-2.

Hence, chronic inflammation has proven to be a key phenomenon in cancer development (Figure 2) since it is closely related to the processes of cellular metaplasia and tumor promotion, survival, proliferation, invasion, and angiogenesis [71]. Chronic inflammation is characterized by inflammatory cell infiltration, such as macrophages, lymphocytes, and plasmatic cells, which together release cytokines (TNF-α, IL-6, TGF-β, and IL-10), growth factors, and enzymes, contributing to tissue damage and repair [72]. However, these mediators’ chronic actions have been associated with tumor initiation and progression [73]. Thus, to connect inflammation with cancer, typically two pathways are described; an intrinsic pathway depends essentially on oncogene activation and tumor suppressor gene silencing (tumor-promoting role). The other extreme is the extrinsic pathway, which is the inflammation resulting from infections or other inflammatory processes that precede cancer development (tumor-initiating role) [74].

On the other hand, an inflammatory environment is prone to produce large amounts of reactive oxygen species (ROS) and reactive nitrogen species (RNS), a group of molecules collectively called RONS, produced by the inducible nitric oxide synthase (iNOS) and NADHP oxidase, resulting in phagocytic cell activation. Moreover, pro-inflammatory cytokines induce RONS production in non-phagocytic cells in the inflamed niche [75,76,77]. Multiple mechanisms have been proposed to explain the RONS and cancer association. The best-studied molecular damage mechanism by ROS and RONS is related to the high reactivity of these molecules against lipids, proteins, and DNA. Thus, when guanine reacts with ROS, 8-oxo-7,8-dihydro-2′-deoxyguanosine (8-oxoDG) originates, a metabolite prone to cause G:C transversion (G➔T transversion) to T:A [78,79]. On the other hand, when 8-oxoDG reacts with ONOO^−^, 8-nitrodeoxyguanosine generates, causing a similar transversion [80,81].

Other critical pathways are STAT3 and NF-κB activation triggered by IL-6 and TNF-α, respectively, indispensable for perpetuating inflammation. NF-κB leads to pro-inflammatory cytokines, chemokines, RONS, and adhesion molecules induction [82] and the activation of cellular proliferation and differentiation pathways, which drives to pro-tumorigenic activity increase [83]. Additionally, it has been associated with therapy resistance, based on reports that persistent activation of the NF-κB pathway is associated with a worse prognosis [84]. Interestingly and controversially, it also holds anti-tumoral properties that lay on the expression of Th1 cytokines, such as IL-12 and IFN-γ [85,86,87]. However, STAT3 overshadowed these anti-tumoral effects, limiting this response by inducing a Th2 response with the release of IL-10, pushing macrophages into the M2 phenotype [88]. Additionally, it contributes to the expansion and development of Treg and Th17 cells, which are linked with tumor growth due to their ability to dissipate anti-tumoral immunity, enhancing both the tumor’s survival and its metastasis capacity [89,90]. In addition, STAT3 pathway activation is tightly related to tumor neovascularization via vascular endothelial growth factor (VEGF) upregulation [91]. Some studies suggest that IL-17 has a vital role in this pathway activation, potentiating the angiogenic effect [92]. Therefore, STAT3 is not only a pro-tumoral agent but also intervenes as an anti-tumoral response inhibitor [93].

During the first steps in neoplasia development, nutrient scarcity forces tumor cells to balance between apoptosis and cellular growth. This pre-angiogenic or “avascular” stage restricts growth and dissemination, reaffirming the angiogenesis importance [94] since when angiogenesis occurs, rapid tumor growth occurs, leading to poorly irrigated regions with a consequent hypoxic and acidic milieu [95], a fact having a profound impact by the VEGF release stimulated by a lower pH and, thus, enhancing angiogenesis [96]. On the other hand, low oxygen concentration stimulates hypoxia-inducible factor production (HIF), another group of angiogenic peptides involved in metabolic reprogramming, invasion, metastasis, and treatment resistance [97]. In summary, aside from stimulating tumor growth and preservation, angiogenesis is also an indispensable factor in tumor invasion and dissemination due to the wide vascular surface available for malignant cells to penetrate without mentioning the increased vascular permeability [98].

From the immunological perspective, immune evasion is a hallmark of cancer [99]. This characteristic is the result of multiple dysregulation phenomena induced by the previously mentioned tumor microenvironment. This situation is known as “tumor-induced immunosuppression”, characterized by an environment where pro-tumor immune mediators are hierarchized over anti-tumor ones: a Th2 inflammatory response dominance, M2 macrophages, Treg cells, a low capability for antigen presentation and cytotoxicity, and cytokines, such as TGF-β, IL-6, IL-8, IL-10, and VEGF [100].

Furthermore, mentioning AA derivatives is a sine qua non condition when discussing inflammation, since many studies suggest that patients who regularly take non-steroidal anti-inflammatory drugs (NSAIDs) have a lower risk of developing a wide range of neoplastic diseases and a lower risk of metastasis [101,102,103]. This affirmation makes more sense after knowing that PGE_2_ is the most abundant prostanoid found in several malignant lesions and that its pro-inflammatory nature contributes to tumor growth [104]. Therefore, interfering with this chain of events is considered one of the new lines of therapy against cancer [105,106,107].

Finally, it is worth mentioning that not all malignancies possess the same immunological behavior. Recent advances have suggested new methods to classify tumors according to the distribution and to quantity of lymphocytes CD3 and CD8, as well as the expression levels of B7-H1/PD-L1 [108]. Consequently, tumors can be divided into “hot”, “intermediate” (including immunosuppressed and isolated), and “cold”. On the one hand, “hot tumors” are highly infiltrated tumors with abundant anti-tumoral cells and positive responses to conventional therapies; on the other hand, “cold tumors” not only do not lack anti-tumoral cells, but are also rich in immunoevasive molecules and, therefore, associated with a worse prognosis. Additionally, other immune coordination profiles have been described for those patterns that do not correspond to the previously explained “hot” vs. “cold” classification. In this regard, the “excluded” and “immunosuppressed” phenotypes were accepted: the first one, to describe tumors with T-cells on the edge of tumoral sites or “invasive margins”—unable to infiltrate the parenchyma; the second corresponds to malignancies with low levels of immune infiltration without any physical barrier, suggesting the existence of an immunosuppressive environment within the tumor that hampers anti-tumoral functions [109,110]. This novel classification, or immunoscore, that allows the characterization of tumors beyond their expansion has been highly validated around the world not only for its greater prognostic value but for the new possibilities that arise with regard to anti-tumoral therapies based on immunology, such as T-CAR cells, immune checkpoint inhibitors, or oncolytic viruses. These are examples of techniques that could be enhanced and personalized according not only to the TNM classification but also to the immunoscore, type of cancer, and many other variables, leading to a new era in cancer therapeutics [111].

## 4. Anti-Tumoral Mechanisms of SPMs: An Approach to Inflammatory Responses

**Figure 3:**
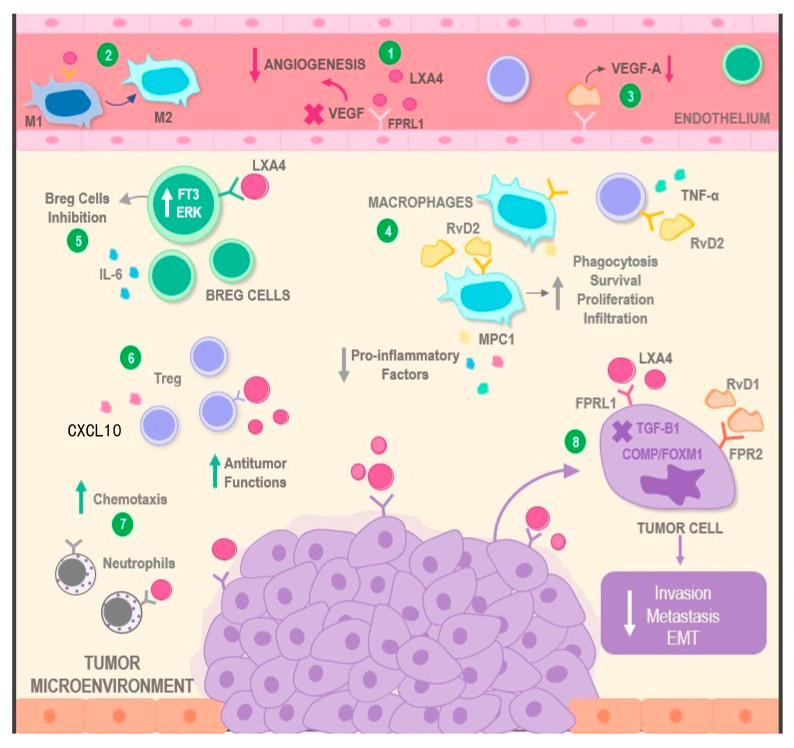
Mechanisms of action involving SPMs in the modulation of the tumor microenvironment. SPMs display multimodal mechanisms of action over malignant neoplasms. On one hand, the binding of molecules, such as LXA4 and RvD1 to the FPRL1 receptor, can result in a decrease in angiogenic phenomena via VEGF inhibition (1 and 3). On the other hand, the interaction between LXA4 and macrophages may restore their anti-tumoral actions via their transition from the M1 towards the M2 phenotype (2). Similarly, RvD2 increases characteristics such as phagocytosis, infiltration, proliferation, and survival of M2, as well as simultaneous reduction of pro-inflammatory cytokines, such as MPC1, IL-6, TNF, and CXCL10, in several types of immune cells located in the TME (4). Additionally, LXA4 can exert inhibitory effects over pro-tumoral cells, such as Breg lymphocytes (5), and stimulant effects on anti-tumoral immune cells, such as Tregs and neutrophils, potentiating their antineoplastic activity (6) or increasing the levels of chemotaxis towards the tumor (7). Finally, both LXA4 and RvD1 directly lead to the decrease of essential pro-tumoral processes, such as invasion, metastasis, and EMT, by interacting with receptors located on the surface of cancer cells (8). Abbreviations: RvD2: Resolvin D2; RvD1: Resolvin D1; SPMs: Specialized Pro-Resolving Mediators; TNF-α: Tumor necrosis factor alpha; IL-6: Interleukin 6; MPC1: Mitochondrial Pyruvate Carrier 1; CXCL10: C-X-C Motif Chemokine 10; LXA4: Lipoxin 4; VEGF: Vascular Endothelial Growth Factor; FT3: Transcription Factor 3; ERK: Extracellular Signal-regulated Kinase; Breg cells: Regulatory B cells; FPRL1: Formyl Peptide Receptor-like 1; FPR2: Formyl Peptide Receptor 2; EMT: Epithelial Mesenchymal Transition; TGF-B1: Transforming Growth Factor Beta 1; COMP: Cartilage Oligomeric Matrix Protein; and FOXM1: Forkhead Box Protein M1.

With better comprehension of the pathways that connect inflammation with cancer, the robust role of SPMs in the resolution of inflammation supposes a modulating mechanism in the transition to cancer. Therefore, the focus of their participation in this process has been widened in recent times, being acknowledged now that these bioactive lipids, via multimodal mechanisms, are capable of influencing diverse aspects of neoplastic development. These range from direct actions on malignant cells to the modification of functions and behaviors of multiple components in the TME (Figure 3), which also influence pro-tumor phenomena that are essential for the evolution of cancer. Hence, it is necessary to approach these potential mechanisms individually [34,37,112]. 

### 4.1. SPMs and the Resolution of Inflammation

Through the collected evidence, it has been established that, for a proper resolution of inflammation, it is indispensable to inhibit the migration and activation of neutrophils toward the site of inflammation. SPMs have this mechanism of action in common, enhancing this phase [113]. At the same time, neutrophil degranulation contributes to the spread of the inflammatory response, which is why LXs exert their effect by inhibiting them [114]. It must also be taken into account that the resolution of inflammation greatly depends on the efferocytosis of apoptotic neutrophils and dead cells during the initial phase of inflammation. This is a prerequisite for effective phagocytosis [115]. Here is where Rvs and LXs come into action by promoting this mechanism effectively and favoring chemotaxis and the adhesion of non-inflammatory monocytes. They stimulate the phenotype switch of pro-inflammatory M1 macrophages into the anti-inflammatory M2 phenotype. This effect is further potentiated by MaRs that regenerate cells and tissues, particularly in certain organs, such as the heart [116,117]. Moreover, beyond its regenerative actions on various tissues, it has been reported that RvD1 exerts powerful suppressing effects over tumoral proliferation through the modulation of classic monocytes transmigration via increased MCP-1 expression in a human papillomavirus (HPV)-positive cancer cell model [118].

Moreover, it is important to add that all SPMs protect from the oxidative stress generated by pathogens and immune cells, not only by reducing ROS and RONS but also by stimulating the expression of antioxidant defenses, such as Superoxide Dismutase (SOD), Hemo-1 Oxidase (HO-1), and Nrf2, which protect against inflammation, liver toxicity, and even cancer [119,120]. Additionally, SPMs can intervene in adaptive immunity, with the recruitment of T CD4^+^ and CD8^+^ cells [121] capable of reducing the synthesis of pro-inflammatory cytokines and preventing the differentiation of T CD4^+^ cells into the TH1 and TH17 subsets, which are related to many diseases, via the regulation of transcription factors, without being immunosuppressive [122]. This fact was confirmed by Chiurchiu et al. [123], who found that DHA-deficient mice had high levels of these cells, and as a result, the supplementation with RvD1, RvD2, and MaR1 significantly reduced the synthesis of pro-inflammatory cytokines due to the stimulation of T cells, thus regulating chronic inflammation. While RvD1 and RvE1 influence the humoral immune response by increasing the synthesis of IgM and IgG on active B cells, they reduce the production of IgE [124]. Studies show that RvD1 expedites allergen clearance in mice models with allergic respiratory diseases, while RvE1 improves the lymphatic clearance of phagocytes and blocks platelet aggregation and activation [57,124].

It is important to point out that a considerable part of the inflammatory response is a result of the activation of transcription pathways, which allow its perpetuation. This is the reason why LXs can regulate several levels of transcription factors, such as NF-κB, in a joint action with MaRs. In addition, they control the expression of several genes linked to inflammation and inhibit the Activator protein 1 (AP-1), nerve-growth-factor-inducible protein A (NGFI-A), and the peroxisome-proliferator-activated receptor γ (PPARs) [112,125]. On the other hand, the actions of NPD1 are involved mainly in neuroinflammation. This is why Bazan et al. [62,126] pointed out that they are modulators of stress pathways that relate to cell death and the increase of cell survival.

Particular approaches have described beneficial SPM effects over tumor-promoting inflammation. These hypotheses have been sustained by the existence of tumoral progression-modulating mechanisms that exert an inflammation-promoting effect over leucocytes via apoptotic cellular debris produced as a consequence of chemotherapy [127,128]. In this context, Sulciner et al. [27] presented the inhibiting effects of RvD1, RvD2, and RvE2 on tumoral progression stimulated by cellular debris. Additionally, they reduced inflammatory responses as a result of the suppression of chemokynes and cytokines, such as TNF-α, IL-6, IL-8, CCL4, and CCL5.

### 4.2. SPMs and the Resolution of Tumoral Angiogenesis

It has been reported that SPMs exert an anti-tumoral effect via the inhibition of uncontrolled angiogenesis. This fact is supported by several experimental models; however, the mechanisms by which these mediators offer their anti-angiogenic activity have not been completely elucidated [129]. LXA4, by binding to its receptor (ALXR), can reduce the phosphorylation of the VEGF receptor (VEGFR) and reduce the synthesis of angiogenic mediators, such as VEGF-C. It can also attenuate the production of inflammatory mediators, such as PGE2, LTB4, IL-6, and IL-8, in malignant cells extracted from a human Kaposi sarcoma tumor [130]. In another underlying mechanism, LXA4 is also capable of diminishing the angiogenic process by lowering the synthesis of HIF-1α and, as a result, significatively reducing tumor growth [131].

For their part, RvD1 and LXB4 also generate a decrease in the expression of mRNA of pro-angiogenic factors and a reduction in VEGF-A release via a mechanism that involves STAT-3 signaling in gastric cancer cell lines. It has also been found that this antiangiogenic effect may be mediated by the activity of the formyl peptide receptor 1 (FPR1), which is a pattern recognition receptor (PRR), after seeing that the pharmacological suppression of this receptor increased the tumoral pro-angiogenic activity and diminished the activity of SPMs, their receptors, and the enzymatic components associated with their production [132]. In addition, an anti-angiogenic effect has been described for RvE1 after finding that, just like RvD1 and the ATL analog, it decreased corneal neovascularization. RvE1 exerted this effect by binding to ChemR21, which is widely expressed in this tissue. However, there is still not enough evidence of the potential effect of RvE1 in tumoral angiogenesis [133,134].

### 4.3. SPMs and Tumoral Immunomodulation

Even though the immune system is considered the natural defense of the body, tumoral cells have effective abilities not only at the time of evading immune responses but also in manipulating cell behavior and redirecting it to favor tumor progression. This way, they convert the immune elements inside the TME into allies responsible for maintaining chronic inflammation and ensuring neoplastic development [135,136,137]. In this context, the role of SPMs in the re-establishment of physiological anti-tumoral functions in immune cells that reside in the TME has been recently discovered to stop the evolution of cancer [32].

Firstly, studies have shown the effects these molecules have on leukocytes, such as neutrophils and PMN, by inhibiting key actions for the preservation of chronic inflammation, such as chemotaxis, migration, epithelial interactions, and the release of pro-inflammatory cytokines from these cells. In addition, they inhibit the synthesis of toxic substances, such as free radicals, which are capable of damaging nucleic acids, causing genomic instability, and, with this, potentially oncogenic mutations. They also promote their anti-tumoral activities by stimulating phagocytosis in PMN and cytotoxic actions in neutrophils [138,139].

Furthermore, the immunomodulatory effects of SPMs extend to other immune cells and, in particular, to macrophages and their precursors. Studies have shown the inhibiting role of the ATL-1 analog in the proliferation of monocytes to reduce the number of potential tumor-associated macrophages (TAMs) and, in this way, decrease the tumoral progression and increase the survival rates in patients [140]. However, similar trials performed in vivo using LXA4 show opposite results and report increases in monocyte levels, their chemotaxis, subsequent differentiation to M2, and preservation of the anti-tumoral phenotype, preventing the “switch” toward tumor-associated macrophages or M1 [141,142,143]. Even beyond maintaining physiologic activities in cells, a potentiation in phagocytic actions has been observed. This is induced by the administration of Rvs, as well as the blocking of apoptosis through mechanisms, such as the activation of the PI3K/Akt pathway. This causes the expression of the Bc12 anti-apoptotic protein, the disruption of the caspase pathway due to the absence of pro-apoptotic stimuli or signals, and the preservation of mitochondrial integrity to decrease the generation of ROS. Overall, these effects indicate a positive impact of these lipids, both in the reduction of malignant phenotypes as well as in the preservation and optimization of anti-tumoral activities [24,27,144].

Lastly, preclinical studies carried out in mice have highlighted the existing relationship between the effects caused by LXA4 and RvD1 in B, T, and NK cells. On the one hand, RvD1 is responsible for maintaining cytotoxic actions in NK cells according to what was proven in vitro, where pancreatic cancer cells were used. Similarly, researchers have listed the promoting effects of LXA4 in lymphocytic cells with altered phenotypes, especially on Treg lymphocytes in the early stages of cancer [145,146]. Contrary to the effect exerted on Tregs, there has been confirmation of suppressive effects of LXA4 on regulatory B lymphocytes (Bregs), which synthesize IL-10, due to the role these have on the promotion of tumoral development because of the negative regulation over other immune cells that they carry out. Therefore, the inhibition of Bregs via mechanisms, such as dephosphorylation of STAT3 and ERK factors by LXA4, results in a decrease in tumoral growth [49].

### 4.4. SPMs and Precancerous Lesions

In the continuous study of carcinogenesis, the changes provided by the inflammatory microenvironment in organs at risk must be mentioned [71]. This substantially contributes to the development of preclinical pathological changes that constitute predisposing factors for the development of an invasive lesion, whose detection is one of the cornerstones in the secondary prevention of neoplasms [147]. In this sense, SPMs offer a protection mechanism to minimize the tissue damage observed in chronic inflammatory disorders and, therefore, decrease the neoplastic transformation [148].

One of the phenomena involved in this event is the epithelial–mesenchymal transition (EMT), described as the differentiation process of epithelial cells towards an undifferentiated or mesenchymal phenotype [149]. It is associated with local tumor invasion, metastasis, premalignancy processes, and fibrosis [150]. In the EMT, there is a loss of the normal cellular structure due to a decrease in the expression of certain factors, such as E-cadherin, and an increase in the expression of vimentin. These have both been postulated as predictors of tumoral behavior and markers for the early detection of some neoplasms [151,152].

Researchers have tried to demonstrate the potential impact of SPMs on this process. In a study by Zong et al. [153], it was reported that LXA4 was capable of reverting EMT after binding to FPRL1. It was used as a treatment in a model with pancreatic cell lines, also evidencing that LXA4 influenced this process via the suppression of TGF-β1 signaling. This resulted in a decrease in local invasion and metastasis. Analogously, this fact has been explored with RvD1, depicting the important role of this mediator in EMT prevention. This was witnessed in co-cultures of hepatocellular carcinoma cells and cancer-associated fibroblasts (CAF), where RvD1 binds to its FPR2 receptor and can repress CAF-mediated EMT in liver cells through the decrease in FOXM1 and COMP expression, subsequently decreasing cellular invasion [154,155].

On the other hand, another precancerous disorder that has been linked with these bioactive lipids is inflammatory bowel disease (IBD), which has been recognized as an important risk factor for the development of CRC and colitis-associated cancer (CAC) [156]. SPMs have a role as preventive agents in this setting, after describing their effect on the resolution of inflammation in the intestinal mucosa in experimental models [157,158]. Several studies have demonstrated that RvE1 and MaR1 have a modulator effect on the inflammatory response in mice models with dextran sulfate sodium (DSS) and 2,4,6-trinitrobenzene sulfonic acid (TNBS)-induced colitis. This evidenced a decrease in the synthesis of several inflammatory mediators, such as IL-1β, TNF-m, IL-6, and IFN-γ, and in the case of MaR1, a decrease in neutrophil recruitment and ROS production was observed. This lowering effect on colon inflammation mediated by both compounds is dependent on the inhibition of NF-κB signaling [159,160]. NF-κB inhibition is also considered a regulating mechanism of progression to CRC since its increased activity promotes the aberrant production of chemokines and tumorigenic proteins responsible for the inhibition of apoptosis and an increase in cellular survival, as previously described [161].

In another order of ideas, researchers have studied the preventive effect of SPMs from the perspective of their synthesis. They have demonstrated that the suppression of 15-LOX-1, which is an important enzymatic regulator of the production of these lipid mediators, increased the incidence of CAC via a mechanism that involves the IL-6/STAT3 pathway. This established the association between the regulation of the activity of SPMs and the development of CAC [162,163].

In the context of hepatic lesions, RvD1 and RvE1 have a protective effect by relieving the liver injury caused by concanavalin A (ConA) in animal models. Pretreatment with RvD1 and RvE1 in C57BL/6 mice in whom liver damage with ConA was induced had a decrease in the production of TNF-α, IFN-γ, IL-2, IL-1β, and IL-6, as well as an inhibition of T CD4^+^ and CD8^+^ lymphocyte infiltration via a mechanism dependent on the inhibition of NF-κB and AP-1. This established the protective role of RvD1 and RvE1 in the progression from hepatitis to liver cancer [164]. The effect has also been attributed to PD1 and MaR1 since these are capable of reducing ConA-induced liver injury and, thus, attenuate the progression of the disease by inhibiting the CX3CL1/CXRCR1 axis and decreasing ROS production, respectively, via a mechanism also dependent on NF-κB [165,166].

Finally, it has been reported that prolonged exposure to ultraviolet rays (UVR) is an important carcinogenic factor by causing disturbances in the antioxidant mechanism of the skin and by triggering morphological changes in it [167]. A recent study exposes the protective effect of MaR1 by promoting the resolution of inflammation and reducing the oxidative stress caused by UVR. The administration of MaR1 reduced cutaneous edema and neutrophil recruitment, inhibited keratinocyte apoptosis, and decreased epidermal thickening and collagen degradation in the skin of hairless mice exposed to UVB radiation [168].

## 5. Beneficial of Supplementation with ω-3 PUFAs in Cancer: Clinical Evidence

In understanding the expanding role of the products of lipid metabolism over the regulation of the inflammatory process and, as a result, on the development of cancer, the modulation of the profile of activity of these mediators represents a therapeutic alternative to study [169,170]. Therefore, in the last few years, supplementation with foods rich in ω-3 PUFAS, such as fish oil, has gained a lot of interest after demonstrating that these lowered the incidence of cancer in several experimental models [171,172,173]. On the basis of this premise, researchers have documented a wide variety of observational studies to demonstrate the role of EPA and DHA as therapeutic and chemopreventive agents for the development of cancer and tumoral progression (Table 1) [174,175]. 

To this end, it is necessary to highlight clinical trials that have sought to explore this fact, in the individual context of each cancer. One has been CCR, for which studies have endeavored to explore the role of ω-3 PUFAs as chemopreventive agents or adjuvant therapy to improve its survival [43]. A study published by Cockbain et al. [176] evaluated the role of ω-3 PUFAs on patients with metastatic CCR before undergoing liver resection surgery. This phase III clinical trial reported that 18 months after surgical resection, the supplementation with EPA-FFA increased the general survival rate (SR), when compared with controls, due to its antiangiogenic properties. Similarly, the CALGB 89803 study retrospectively studied the relationship between consumption of marine-derived ω-3 PUFAs and survival in patients with completely resected stage III CCR. It showed that this supplementation increased the rate of disease-free survival (DFS), revealing that patients who consumed dark fish at least one day a week had a 35% lower risk of recurrence of death by cancer [177].

On the other hand, a multicentric study (The seAFOod Polyp Prevention Trial) evaluated the potential effect of treatment with EPA and aspirin, on their own or in combination, for the prevention of CCR. To achieve this, four groups were assigned; two of them received individually 2 g/day of EPA (as FFA or TAG) or 300 mg/day of aspirin. Another group received both simultaneously, and the last group was administered a placebo for 12 months. After the follow-up colonoscopy, results showed that the supplementation with EPA and aspirin did not reduce the ratio of individuals with one or more colorectal adenomas. However, it was established that both confer certain chemoprevention against adenoma, so this must be evaluated in the future with precision medicine [178]. Moreover, results are expected from the OMICC study (NCT03661047), which seeks to evaluate the effect of ω-3 PUFAs in the treatment with AMR101 (VASCEPA, icosapent ethyl) in the tumor microenvironment and gut microbiota in patients diagnosed with colon cancer.

Within the context of prostate cancer, there is a lot of evidence regarding the regulator effect of ω-3 PUFAs on this disease [179]. A clinical trial performed by Aronson et al. [180] explored the role of the low-fat fish oil (LFFO) diet with a reduction in the ratio of ω-6 to ω-3 in individuals subjected to radical prostatectomy. The participants consumed five capsules of fish oil (1.1 g each) per day during the study. It was evidenced that the LFFO diet did not provide statistically significant changes in the profile of biomarkers, such as IGF-1. However, secondary results showed in the Ki67 immunostaining a reduction in the proliferation of malignant tissue of 32.2% (*p* < 0.05). This proved the inhibitory effect of the LFFO diet in carcinogenesis and prostate cancer progression. Later, a study derived from this trial assessed the effect of this diet on the profile of serum eicosanoids. There was a decrease in the profile of 15 (S)-HETE and the cell cycle progression score (CCP) with the LFFO diet. The 15 (S)-HETE/LTB_4_ pathway was highlighted as a potential target in the proliferation of this type of cancer [181]. Currently, results are expected from a phase IIb trial that is evaluating the effect of supplementation with MAG-EPA over cellular proliferation, inflammation, and quality of life in patients with prostate cancer and a Gleason score ≥7 who are about to undergo a radical prostatectomy [182].

Another cancer that has been studied for the effects of PUFAs both in the therapeutic and preventive field is breast cancer [183]. Within the evidence, a small clinical trial stands out, where they explored the effect of supplementation with 2 g/day of fish oil over the nutritional and immunological parameters of patients with breast cancer without treatment, with previous chemotherapy. These results showed that supplementation led to an increase in serum levels of EPA (*p* = 0.004) and DHA (*p* = 0.007), also evidencing the preservation of serum levels of high-sensitivity C-reactive protein (hsCRP) and T CD4^+^ lymphocytes, showing signs of a modulator effect of ω-3 PUFAs on the immune response and inflammation of these patients [184]. In addition, the potential of DHA as neoadjuvant therapy in metastatic breast cancer has been reported. It improves the result of chemotherapy by increasing the tumor’s sensitivity to the treatment and, as a result, survival [185].

Based on this potential, a clinical trial was performed on 48 patients with locally advanced breast cancer. The intervention consisted of the administration of ω-3 PUFAs supplements (1 g/d) or placebo, along with the administration of three cycles of neoadjuvant chemotherapy with cyclophosphamide-doxorubicin-5′fluorouracil (CAF). It was then demonstrated that the supplementation significantly improved the general survival of patients when compared with controls. Moreover, there was a decrease in the expression of Ki67 (*p* = 0.032) and VEGF (*p* = 0.041), exposing the benefits of supplementation with ω-3 PUFAs in neoadjuvant chemotherapy for this type of cancer. Nevertheless, other variables must be explored further, such as the optimal dose, bioavailability, and circulating levels in future investigations [186]. On the other hand, results are expected from the DHA-WIN (NCT03831178) study, which has the objective of evaluating the potential therapeutic index (efficacy: adverse effects) of supplementation with 4.4 g/d of DHA in combination with neoadjuvant chemotherapy in patients with early breast cancer, using the Ki67 index as a marker for efficacy as well as the effect of DHA over serum phospholipids and the immune system [187]. Lastly, it is essential to mention that, even though there is an ample bibliography on the beneficial role of ω-3 PUFAs and cancer development, these results are not conclusive and must be interpreted with caution, as there are also studies showing a weak association between ω-3 PUFA supplementation and cancer suppression in certain types of cancer [188].

**Table 1:** Summary of clinical evidence for the treatment of EPA and DHA in different types of cancer.

Type of Cancer	Methodology	Intervention	Relevant Results	Ref.
Colorectal cancer	Phase III, randomized, double-blind, placebo-controlled study in 88 patients with CRCLM before undergoing liver resection surgery	Dietary supplementation with 2 g/day of EPA-FFA during the preoperative period compared with placebo.	The daily consumption of EPA-FFA increased the OS rate in patients 18 months after the surgical intervention. There were no differences in Ki67 PI, but the antiangiogenic activity of EPA-FFA was proven in vitro	[176]
	Retrospective study on 1011 patients with stage III colon adenocarcinoma, without metastasis, prior chemotherapy, or radiotherapy.	Marine-derived ω-3 PUFAs ingestion (g/d) was calculated by multiplying the portion of each article, the frequency, and the sum of all the articles.	People who consumed dark fish ≥1 d/week obtained a higher DFS rate than those who did not (HR 0.65; CI of 95%, 0.48 to 0.87; *p* = 0.007).	[177]
	Multicentric, randomized, double-blind, placebo-controlled, two-by-two factorial study in 709 patients with sporadic CRC diagnosed with colonoscopy	The 4 study groups received: (1) 2 g/day of EPA-FFA or EPA-TAG; (2) 300 mg/day of aspirin; (3) Aspirin and EPA; or (4) Placebo	Supplementation with EPA and aspirin did not decrease the percentage of individuals with one or more colorectal adenomas. There is a need for more evidence with precision medicine.	[178]
Prostate cancer	Phase II randomized study on 48 patients with localized prostate cancer, who were about to undergo radical prostatectomy. LFFO assessed for 4/6 weeks.	Subjects were assigned to 2 groups: (1) WD and a 15:1 proportion of ω-6:ω-3 PUFAs or (2) LFFO diet with a 2:1 proportion of ω-6:ω-3 PUFAs. 5 cap/day	The LFFO diet did not provide changes in biomarkers, such as IGF-1. However, the activity of Ki67 was reduced by 32.2%, demonstrating an effect on cell proliferation.	[180]
	Post hoc analysis that used serum and prostate tissue samples obtained in a previously completed randomized clinical trial.	The levels of LBT_4_ and 15 (S)-HETE were assessed, as well as CCP score. FA was measured with GC and ELISA.	Levels of ω-6 PUFAs and 15 (S)-HETE were significantly decreased in comparison with controls. 15 (S)-HETE was correlated with Ki67 (*p* < 0.01). CCP score was significantly lower in individuals with the LFFO diet than those with WD.	[181]
Breast cancer	Randomized, double-blind, clinical trial that evaluated the effect of ω-3 PUFAs over immune parameters in 45 patients with breast cancer without chemotherapy.	Provided 2 g (470 mg of EPA and 390 mg of DHA) of fish oil concentrate each day (2 caps/day) at lunch and dinner time for 30 days.	Supplementation with ω-3 PUFAs resulted in a significant increase in EPA (*p* = 0.004) and DHA (*p* = 0.007) plasma levels. The level of hsCRP and the percentage of CD4^+^ lymphocytes were maintained when compared with controls.	[184]
	Randomized, double-blind, placebo-controlled clinical trial in 48 patients with locally advanced breast cancer treated with neoadjuvant CAF.	Administered 1 g of ω-3 PUFAs each day, along with 3 cycles of neoadjuvant chemotherapy with CAF for 51 days.	A significant decrease was observed in the expression of Ki67 and VEGF when compared with controls. In addition, OS and DFS rates were significantly higher: *p* = 0.048 and *p* = 0.044	[186]

Abbreviations: CRCLM: Colorectal cancer with liver metastasis; ω-3 PUFAs: omega-3 Polyunsaturated fatty acids; EPA: Eicosapentaenoic Acid; DHA: Docosahexaenoic Acid; EPA-FFA: Eicosapentaenoic Acid-Free Fatty Acid; OS: Overall survival; DFS: Disease-Free Survival; hsCRP: high sensitivity C-reactive protein; 15 (S)-HETE: 15-Hydroxyeicosatetraenoic acid; CCP: cell cycle progression; WD: Western diet; LFFO: Low-fat fish oil; CRC: Colorectal cancer. CAF: Cyclophosphamide, Doxorubicin, 5-Fluorouracil; and GC: Gas chromatography.

## 6. Conclusions

Ever since the identification of the hallmarks of cancer, the study of inflammation has gained great relevance. A considerable number of studies have highlighted the important role of inflammation as a promotor of carcinogenesis, an event where the activation of innate and adaptive immunity allows the pivotal participation of diverse types of cells and pro-inflammatory mediators that stimulate each of the stages of tumorigenesis [20,189]. This is accompanied by the elucidation of the protective actions of SPMs, the lipidic mediators that can regulate and suppress the activity of inflammatory agents, neoplastic transformation inductors, and tumoral progression. As a result, SPMs are now an attractive therapeutic target to consider in the journey of improving conventional therapy and decreasing adverse effects.

In this sense, multiple experimental studies have explored the beneficial role of SPMs in ω-3 PUFAs supplementation as a coadjuvant alternative in cancer therapy. As a result, diminished inflammation and risk of metastasis has been observed, as well as improved survival rates in patients, independent of the cancer type. However, despite substantial evidence obtained in the past years, the results remain controversial; thus, more information regarding this topic is required before introducing these supplements as a therapeutical coadjuvant and chemopreventive option in various types of cancer.

## Data Availability

Not applicable.
